# THE EFFECT OF OLDER ADULTS’ STRESS AND COPING ON LIFE SATISFACTION

**DOI:** 10.1093/geroni/igz038.1149

**Published:** 2019-11-08

**Authors:** Meeryoung Kim

**Affiliations:** Daegu University, Daegu, Korea, Republic of

## Abstract

Older adults experience stressors in everyday life, which can be acute or chronic stressors. When people are stressed, coping abilities and social support are important factors for increasing their life satisfaction. Using Pearlin et al.’s (1990) stress process model, this study compared whether acute or chronic stressors were more stressful. Additionally, the effectiveness of emotional, informational and instrumental support on life satisfaction were compared. This study used the 3rd and 5th addition wave of KReIS (Korean Retirement and Income Studies) which were collected in 2009 and 2014 respectively. For socio economic status, 3rd wave data was used. Independent and dependent variables were derived from 5th addition wave data. The sample size of this study was 4,072 older adults who were 65+. Daily hassles were used to indicate acute stressors, and physical and economic strain were used to indicate chronic stressors. For coping resources, social support and coping were used. Life satisfaction was used for dependent variable. Since the stress model is a process model, hierarchical multiple regression was used. Both acute and chronic stressors had a significant effect on reducing life satisfaction. Coping and social support both had significant positive effects on life satisfaction. In regard to social support, emotional and instrumental social support had significant effects on life satisfaction. These results implied that chronic stressors were more stressful than acute stressors. In addition, instrumental social support was found to be better than emotional social support for increasing life satisfaction for Korean older adults.

